# Dendritic calcium spikes are clearly detectable at the cortical surface

**DOI:** 10.1038/s41467-017-00282-4

**Published:** 2017-08-17

**Authors:** Mototaka Suzuki, Matthew E. Larkum

**Affiliations:** NeuroCure Cluster of Excellence, Department of Biology, Humboldt University, Charitéplatz 1, 10117 Berlin, Germany

## Abstract

Cortical surface recording techniques such as EEG and ECoG are widely used for measuring brain activity. The prevailing assumption is that surface potentials primarily reflect synaptic activity, although non-synaptic events may also contribute. Here we show that dendritic calcium spikes occurring in pyramidal neurons (that we showed previously are cognitively relevant) are clearly detectable in cortical surface potentials. To show this we developed an optogenetic, non-synaptic approach to evoke dendritic calcium spikes in vivo. We found that optogenetically evoked calcium spikes were easily detectable and had an unexpected waveform near the cortical surface. Sensory-evoked dendritic calcium spikes were also clearly detectable with amplitudes that matched the contribution of synaptic input. These results reveal how dendritic calcium spikes appear at the cortical surface and their significant impact on surface potentials, suggesting that long-standing surface recording data may contain information about dendritic activity that is relevant to behavior and cognitive function.

## Abstract (web summary)

Surface EEG recordings are thought to primarily detect synaptic activity. Here the authors devise an optogenetic method to evoke dendritic calcium spikes in layer 5 pyramidal cells of the rat somatosensory cortex, and report that optogenetically evoked, as well as sensory-evoked dendritic calcium spikes make a significant contribution to surface EEG recordings.

## Introduction

For nearly a century, brain surface recording techniques such as electroencephalogram (EEG) and electrocorticogram (ECoG) have been the most widely used technique for recording brain activity^1^. The standard textbook explanation of surface potentials is that they primarily reflect cooperative postsynaptic activity^2^ because synaptic currents are assumed to be the dominant electrical current source^3–5^ (but see ref. ^6^ as an example of non-synaptic EEG component). However, other current sources, such as the active properties of cortical pyramidal cell dendrites could potentially contribute^5, 7^. For instance, voltage-gated calcium (Ca^2+^) channels around the main bifurcation point of apical dendrites (550–900 μm from the soma in rats^8^) give rise to characteristic depolarizing plateau potentials (dendritic Ca^2+^ spikes)^9^. These dendritic Ca^2+^ spikes can be generated spontaneously or by sensory inputs^10^, last for 50 ms in anesthetized animals^8^ and up to several seconds in awake behaving rats^11^, and represent a large current source^12^. A recent study further demonstrated the correlation between dendritic Ca^2+^ spikes and animal behavior^13^. Despite their magnitude, functional significance, and proximity to the cortical surface, it remains unclear how dendritic spikes contribute to surface potentials, although it has been observed that dendritic spikes can influence the local field potential (LFP)^7, 10, 14, 15^. We sought to determine the extent to which dendritic Ca^2+^ spikes contribute to the surface potentials through combined use of multi-channel extracellular recordings, optogenetics, pharmacology, and a “micro-periscope” that allows layer-specific light delivery.

## Results

**Fig. 1:**
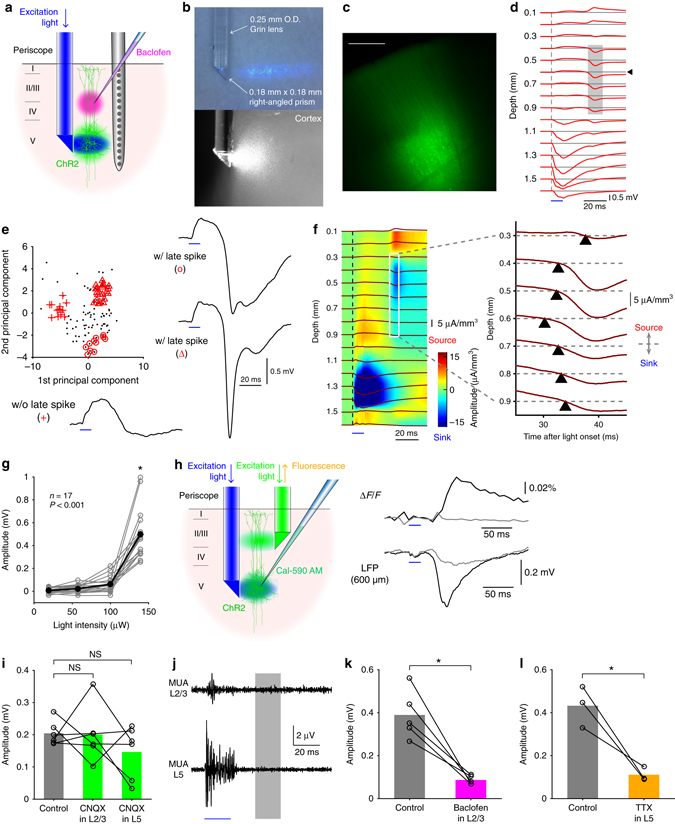
Optogenetically evoked dendritic Ca^2+^ spikes in the local field potential. **a** Schematic diagram of the experiment. **b** A photomicrograph of the micro-periscope system with collimated light in the air (*top*) and in the cortical tissue (*bottom*). **c** A cortical slice showing the virally transfected region co-expressing Channelrhodopsin 2 (H134R) and eYFP. *Scale bar* represents 500 μm. **d** Optogenetically evoked potentials at 16 cortical depths with the highest light intensity (12 mW/mm^2^), averaged over 100 traces in one animal. *Shaded area* indicates the late sink. Blue bar indicates the timing of L5 light stimulation. **e** Principal component analysis of 100 waveforms (*top left*) recorded at 600 μm below the pia (indicated by the *arrow head* in **d**) revealed three types of waveforms: those without discernable late spikes (*bottom left*); those with broad spikes (*top right*); those with huge-amplitude, sharp spikes (*bottom right*). **f** Current source density (CSD) analysis of the evoked potentials averaged over 100 measurements (*left*) and the expanded view between 300 and 900 μm below the pia showing that the late sink initiated at 600 μm below the pia and propagated both upward and downward. **g** The late sink depends on light intensity (one-way ANOVA, *p* < 0.001, *n* = 17 animals). Post hoc multiple comparison test indicates that the amplitude upon highest light intensity significantly differed from others (all *p* < 0.001). **h** Schematic diagram of Ca^2+^ fluorescence imaging experiments (*left*). The sharp increase in Ca^2+^ fluorescence measured in L2/3 coincided with the Ca^2+^ spikes measured with Michigan probe at 600 μm below the pia (*right*). *Black* and *gray traces* are the average of 10 representative measurements with and without discernable Ca^2+^ spikes respectively. The light intensity for L5 stimulation was highest (12 mW/mm^2^). **i** The late sink was unaffected by local application of the synaptic blocker CNQX (100 μM) in L2/3 and L5 (both *p* > 0.05, Wilcoxon signed rank test, *n* = 6). **j** Multi-unit activity (MUA) in L2/3 and L5 after optogenetic stimulation of L5 (averaged over *n* = 5 animals), at the highest light intensity (12 mW/mm^2^). *Shaded area* indicates the period when the late sink was evoked. *Blue bar* indicates the timing of L5 light stimulation. **k** The late sink significantly decreased by local application of 50 μM Baclofen in L2/3 (*p* < 0.05, Wilcoxon signed rank test, *n* = 5). **l** The late sink also significantly decreased by local application of 3 μM TTX in L5 (*p* < 0.05, Wilcoxon signed-rank test, *n* = 3)

Using a linear array of 16 electrodes (Michigan probe), we recorded the extracellular local field potentials (LFPs) in the hindlimb area of the primary somatosensory cortex in the anesthetized rats, while optogenetically stimulating layer 5 (L5) of the cortex (Fig. 1a). Layer-specific light delivery was achieved with a micro-periscope consisting of a 0.18 mm x 0.18 mm micro right-angled prism, a custom-designed Grin lens, and a multi-mode optical fiber (Fig. 1b and Supplementary Fig. 1). Microinjection of adeno-associated virus conjugated with channelrhodopsin-2 (ChR2) and a CaMKIIα promoter to L5 was used to restrict expression of ChR2 predominantly to L5 pyramidal neurons^16^. The fluorescence image of the cortical slices taken from the recorded rats showed the confined viral expression in L5 (Fig. 1c).

### A novel method to evoke dendritic Ca^2+^ spikes in vivo

Ca^2+^ spikes can be evoked in L5 pyramidal neurons by depolarizing the apical dendritic initiation zone^17^. Light pulses delivered to L2/3 caused a sink in these layers, however we chose not to use this approach because it was difficult to separate the effect of the ChR2 current from activation of voltage-sensitive postsynaptic currents. We therefore chose a second method for evoking dendritic Ca^2+^ spikes based on the “critical frequency” approach that uses high-frequency backpropagating action potentials (bAPs) generated at the soma^18, 19^. This method has been shown to activate the same dendritic Ca^2+^ channels as direct dendritic depolarization in vitro^20^ and has been shown to be effective in vivo^21^. By activating ChR2 in L5, we avoided the confounding influence on the LFP that would otherwise have occurred by direct activation of ChR2 in the dendrites (i.e., via light to L2/3). In other words, we could remotely activate the L5 dendrites without directly stimulating the apical dendrites either optically or synaptically.

A single pulse of light (10–30 ms) delivered to L5 reliably caused a current sink in L5 in all cases (Fig. 1d) but also, in 67.8 ± 23.7 (mean ± SD, *n* = 17 animals) % of trials with maximum light amplitudes, we observed an additional sink in the upper and middle layers at distances corresponding to the typical location of dendritic Ca^2+^ spikes^8^ ~ 20 ms after the offset of the light stimulation^18^. Interestingly, the current sink in L2/3 was reliably accompanied by a current source in L1 (Fig. 1d, f and Supplementary Fig. 2). The late sink in L2/3 was sometimes “simple”, and sometimes “complex” in form (Fig. 1e, right) and had variable amplitudes (0.48 ± 0.05 mV, *n* = 17 rats). In the remaining cases, there was no discernable late sink at all (Fig. 1e, bottom left). Current source density (CSD) analysis of the evoked potentials revealed that the second sink was initiated at 613 ± 27 μm below the pia and propagated in both directions, upward and downward (Fig. 1f). The sink was also highly dependent on stimulus strength with a sharp increase occurring at higher light amplitudes (*p* < 0.001, one-way ANOVA with post hoc multiple comparison, *n* = 17 animals, Fig. 1g and Supplementary Fig. 3).

The sink in L2/3 therefore bore all the hallmarks of a dendritic Ca^2+^ spike including location, timing and a sharp dependence on the strength of somatic activation. To investigate this possibility, we performed a number of tests. First, we repeated the optogenetic stimulation experiments combined with a second micro-periscope for recording Ca^2+^ fluorescence in L2/3 (Fig. 1h, left). For these experiments, a Ca^2+^ indicator (Cal-590 AM) with a long excitation wavelength (550 nm) to avoid direct activation of ChR2 was injected to L5. The light through this micro-periscope was shone tonically throughout the experiment so that any residual effect was not time locked to the ChR2 stimulation in L5. Here, optogenetic activation of L5 caused an all-or-none fluorescence transient in L2/3 (at higher stimulus strengths) similar to the current sink seen earlier (Fig. 1h, right). Here the term all-or-none is used to describe the fact that the late sink, while it varied in amplitude and duration, was entirely absent in a significant number of cases resulting in a bimodal amplitude distribution (Supplementary Fig. 4). This is consistent with the late sink correlating with a thresholded event such as a dendritic Ca^2+^ spike. This Ca^2+^ transient was dendritic because the indicator, applied in L5, could only have reached L2/3 by diffusing along the apical dendrites of L5 cells^22, 23^ which suggested that the current sink seen earlier also corresponded to dendritic Ca^2+^ currents. However, to rule out contribution from other postsynaptic elements such as disynaptic firing of cells in L2/3 we injected 6-cyano-7-nitroquinoxaline-2,3-dione (CNQX, 100 μM) into L2/3 and L5. In these experiments, there was no change in the amplitude of the current sink in L2/3 (both *p* > 0.05, Wilcoxon signed rank test, Fig. 1i and Supplementary Fig. 3). We also measured multi-unit activity (MUA) in both L2/3 and L5 (Fig. 1j), which did not show any significant increase of MUA before the onset of the late sink compared with the prestimulus period (all *p* > 0.05, Student’s paired *t*-test, *n* = 5 animals). We therefore conclude that the late current sink in L2/3 was produced by currents in the dendrites of L5 pyramidal neurons.

Previous studies have shown that dendritic Ca^2+^ spikes are effectively blocked by the GABA_B_ receptor agonist baclofen^24, 25^ due to down-regulation of Ca_v_1 (L-type) channels^12^. Here, we found that the dendritic current sinks significantly decreased by local injection of 50 μM baclofen to L2/3 (from 0.39 ± 0.05 to 0.09 ± 0.01 mV, *n* = 5, *p* < 0.05, Wilcoxon signed rank test) (Fig. 1k and Supplementary Fig. 5). Local injection of 3 μM TTX into L5 which would be expected to block APs in the L5 pyramidal neurons also significantly decreased the dendritic current sinks (from 0.43 ± 0.06 to 0.11 ± 0.02 mV, *n* = 3, *p* < 0.05, Wilcoxon signed rank test; Fig. 1l and Supplementary Fig. 6). The late dendritic current sink was clearly not due to bAPs in the dendrites, because they would be expected to take only 1.5 ms to propagate 1 mm back along the dendrites in vivo at 0.67 m/s^14^. Taken together, we conclude that the current sink observed in the middle to upper layers was due to dendritic Ca^2+^ spikes in L5 pyramidal neurons.

### A Ca^2+^ spike-evoked passive current source in L1

**Fig. 2:**
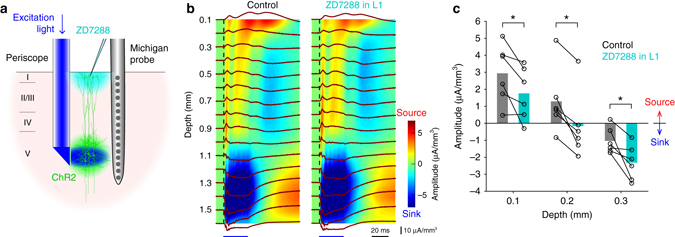
*I*_*h*_ contributes to the generation of passive current in L1. **a** Schematic diagram of the experiment. **b** CSD profiles of evoked potentials before (*left*) and after application of 500 μM ZD7288 to the cortical surface (*right*), averaged over 100 measurements. **c** Summary of the CSD amplitude at 100 to 300 μm below the pia before and after ZD7288 application at the cortical surface (all *p* < 0.05, Wilcoxon signed-rank test)

**Fig. 3:**
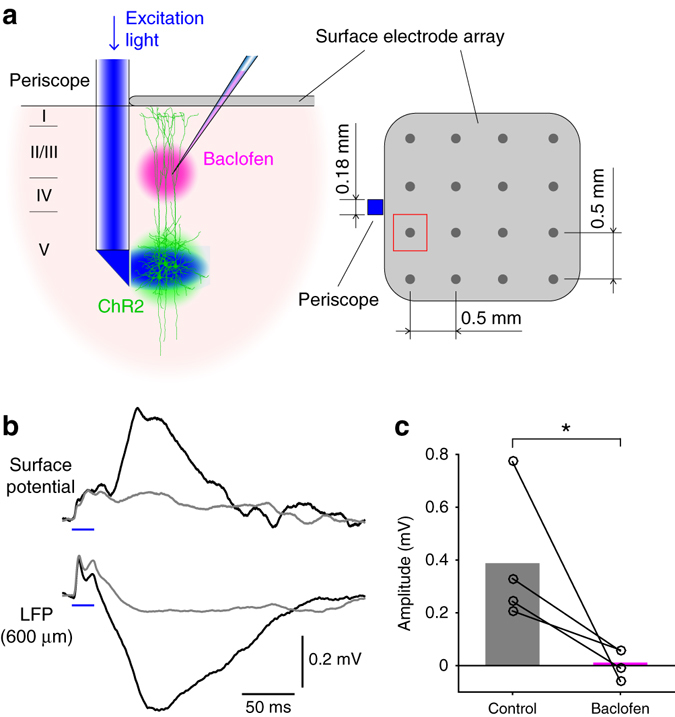
Optogenetically evoked dendritic Ca^2+^ spikes greatly affect surface potentials. **a** Schematic diagram of the experiment. Surface potentials recorded at the electrode close to the micro-periscope (marked in *red*) were analyzed. **b** Surface potentials generated by optogenetically evoked Ca^2+^ spikes (*top*) aligned with LFPs recorded at 600 μm below the pia (*bottom*). *Black* and *gray traces* (average of 10 measurements) are with and without discernable Ca^2+^ spikes, respectively. **c** Average surface potentials before and after application of 50 μM baclofen into L2/3 (*n = *4, *p* < 0.05, Wilcoxon signed-rank test)

**Fig. 4:**
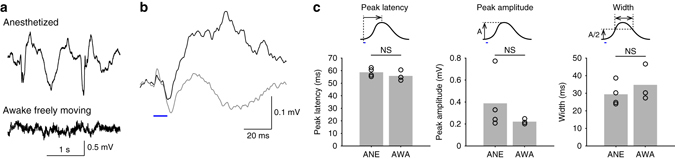
Optogenetically evoked surface potentials in the awake brain. **a** In agreement to numerous previous studies, the surface potential recorded in the awake brain shows high-frequency components compared with the anesthetized brain. **b** Average trace of 50 measurements of surface potential with (*black*) and without the late spike (*gray*). *Blue bar* indicates the timing of L5 light stimulation. **c** Summary of characteristics in the anesthetized and awake brain (all *p* > 0.05, Wilcoxon rank sum test, 4 anesthetized animals vs 3 awake animals)

The appearance of a passive current source in the superficial layers correlating with the Ca^2+^ spike sink in L2/3 was an unexpected finding. Several studies have observed that Ca^2+^ spikes originate in the apical trunk dendrite and rarely propagate to the most distal tuft branches^8, 10, 13, 26, 27^. Furthermore, the distal tuft branches have a high density of the non-selective cation channel (*I*_*h*_)^19, 28–30^ that is spontaneously open, and previous in vitro studies showed that *I*_*h*_ interacts with dendritic Ca^2+^ spikes^31–33^. This spontaneous leak current may account for the pronounced source of passive current. We tested this possibility by local application of *I*_*h*_ blocker ZD7288 (500 μM) on the cortical surface (Fig. 2a). After ZD7288 application the second sink was elongated upward and the source was significantly reduced in both size and duration (all *p* < 0.05, Wilcoxon signed rank test, Fig. 2b, c), suggesting that *I*_*h*_ enhanced the passive current under control conditions. By similar reasoning, we hypothesized that the source near the cortical surface produced by currents in the distal tuft dendrite should also lead to positively deflected potentials at the cortical surface. Using a 4 by 4 surface electrode array (Fig. 3a), we detected surface positive potentials that were 20–30 ms delayed after the onset of the dendritic Ca^2+^ spikes measured at 600 μm deep below the pia (Fig. 3b). In all animals (*n* = 4) the evoked surface potentials were clearly detected at multiple electrodes that were 500 μm apart. The surface potentials were also baclofen-sensitive (from 0.39 ± 0.13 to 0.01 ± 0.03 mV, *n* = 4, *p* < 0.05, Wilcoxon signed rank test, Fig. 3c) and clearly detectable even in the awake freely moving animals (*n* = 3) with similar characteristics (Fig. 4a–c, all *p* > 0.05, Wilcoxon rank sum test). We conclude that apical dendritic Ca^2+^ spikes are detectable as positive potentials at the cortical surface in both anesthetized and awake brain.

### Sensory-evoked dendritic Ca^2+^ spikes

**Fig. 5:**
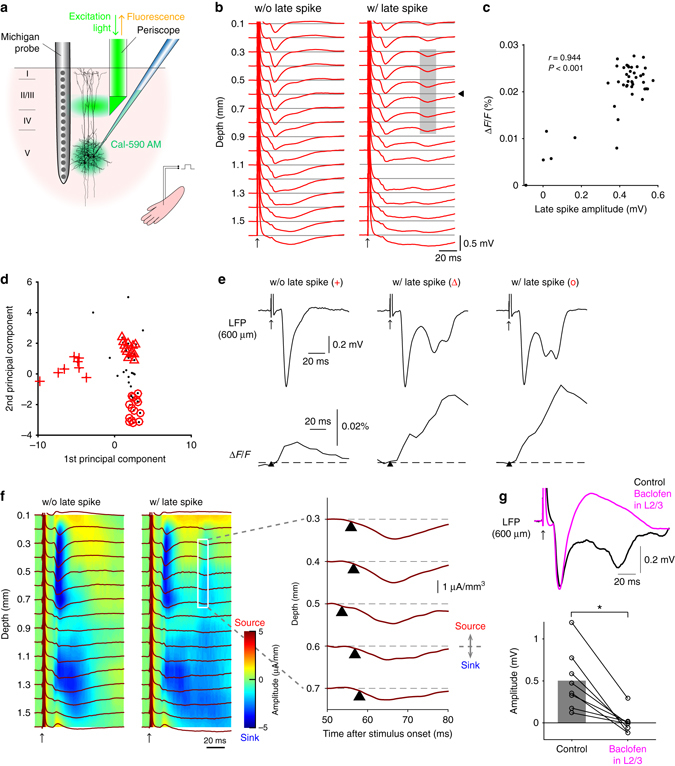
Sensory-evoked dendritic Ca^2+^ spikes. **a** Schematic diagram of the experiment. **b** Sensory-evoked potentials with or without late sink. The late sink was considered present if the peak amplitude exceeded 3× s.d. of the prestimulus activity. Shaded area indicates the late sink. The late sink was present in all rats (*n* = 8). **c** Correlation between the LFP amplitude of the late sink at 600 μm below the pia and dendritic Ca^2+^ transients (*p* < 0.05 in all animals). **d** Principal component analysis of 100 waveforms recorded at 600 μm below the pia (indicated by arrow head in **b**) in a representative animal. **e** Simultaneously recorded LFPs (*top*) and Ca^2+^ transients (*bottom*). From *left* to *right*: those without discernable late sink; those with smaller late sink; those with larger late sink (from part **d**). **f** CSD profiles of sensory-evoked potentials with (*left*) or without (*middle*) the late sink (from part **b**) and the expanded view of the CSD profile between 300 μm and 700 μm showing that the late sink was initiated at 500 μm below the pia and then propagated both upward and downward (*right*). **g** The late sink (*black*, average over all 100 traces) was abolished by 50 μM baclofen (*magenta*, average of 100 traces) shown in a single case (*top*) and for all rats (*bottom*; *p* < 0.05, Wilcoxon signed-rank test, *n* = 8)

**Fig. 6:**
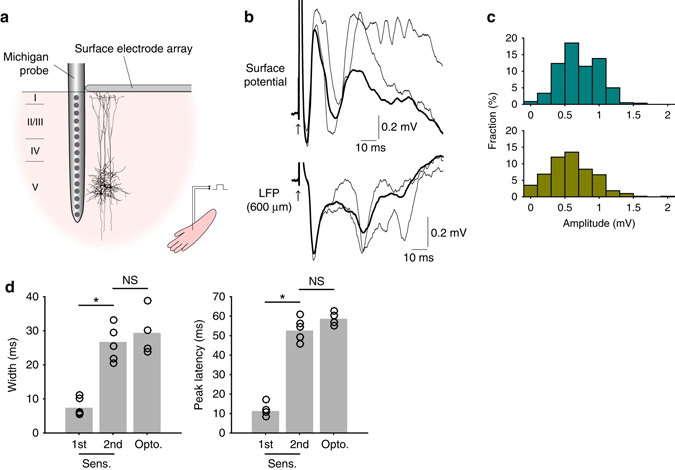
Sensory-evoked dendritic Ca^2+^ spikes also greatly affect surface potentials. **a** Schematic diagram of apparatus for recording sensory-evoked surface potentials. **b** Surface potentials aligned with LFPs recorded at 600 μm below the pia. *Thick lines* denote the average over 10 measurements; *thin lines* show representative traces where the late sink is as large as the first. **c** Surface positive potentials evoked by the first sink (*top*) and those evoked by the late sink (*bottom*). Pooled data from all rats (*n* = 6). **d** Width and peak latency of the first and second spikes evoked by sensory stimulus, compared to optogenetically evoked late spike

**Fig. 7:**
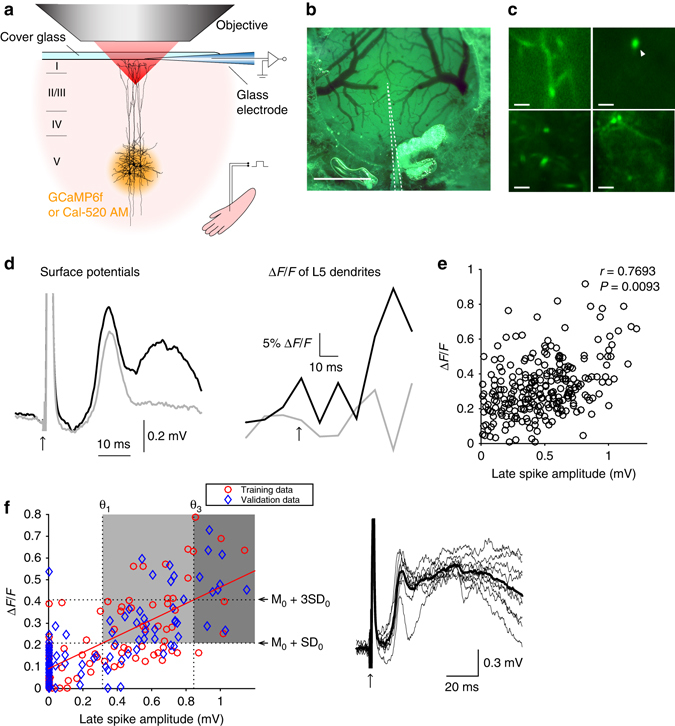
Two-photon Ca^2+^ imaging of individual dendrites and simultaneous recording of surface potentials. **a** Schematic diagram of the experiment. **b** *Top* view of the craniotomy. The *dotted lines* outline the recording glass electrode under the cover glass. *Scale bar* represents 1 mm. **c** Snapshots of imaged dendrites of L5 pyramidal neurons. *Scale bars* represent 10 μm. **d** Simultaneously recorded surface potentials (*left*) and Ca^2+^ transients (*right*) in the dendrite pointed by the arrow head in **c**. *Black* and *gray* traces (average of 20 measurements) are with and without discernable late spikes, respectively. **e** Strong correlation between the amplitude of surface potential and peak fluorescence change during the late spike (*n* = 38 dendrites from four animals). **f** Distribution of training and validation data points from correlated (*r* > 0.8) paired measurements (*left*). A lower threshold θ_1_ of 73.9% (34/46) accuracy was derived from the intersection of the horizontal line (M_0_ + SD_0_) with the *fitted line* to the training data (M_0_ and SD_0_ are, respectively, the mean and the standard deviation of the fluorescence change when surface potentials are zero). A higher threshold θ_3_ of 100% (10/10) accuracy was derived from the intersection of the horizontal line (M_0_ + 3SD_0_) with the fitted line. The classifier was applied to the validation data (*blue*). *Right*: the mean surface potentials in the *dark gray* area (*thick line*) and individual traces (*thin lines*)

Having determined the impact of dendritic Ca^2+^ spikes on surface potentials in the absence of synaptic input to the dendrite (i.e. evoked by optogenetic stimulation of L5), we turned our attention to the effect of *sensory-evoked* Ca^2+^ spikes on surface potentials. We recorded LFPs, surface potentials, and Ca^2+^ fluorescence in the same cortical area—the hindlimb area of the primary somatosensory cortex—while electrically stimulating the contralateral hindlimb (Fig. 5a). Simultaneous recording of LFPs and Ca^2+^ fluorescence revealed two distinct patterns of activity in response to hindlimb stimulation (Fig. 5b). The first kind of response showed a current sink across L2/3 and L5 approximately 10 ms after the stimulus onset, which corresponded to excitatory postsynaptic potentials (EPSPs) as shown in previous studies^34–36^. The first current sink was correlated with a small Ca^2+^ transient observed with the dendritic micro-periscope (Fig. 5e, left). The second type of response arrived ~ 50–60 ms after the stimulus and showed a second current sink in the middle and uppers layers with a similar amplitude and timing to the late Ca^2+^ spike-evoked sink produced by optogenetic stimulation of L5 (Fig. 5b, right). Moreover, the sensory-evoked late sink was accompanied by a large increase in dendritic Ca^2+^ signal (Fig. 5c, e) also similar in amplitude and duration to the optogenetically evoked dendritic Ca^2+^ transient. A principal component analysis of the LFP waveform (Fig. 5d) revealed that the second type of response could be further partitioned into simple or complex waveforms, with the complex waveforms accompanying larger dendritic Ca^2+^ transients (Fig. 5e, middle & right). Consistent with optogenetically evoked dendritic Ca^2+^ spikes, the second current sink was initiated at 538 ± 57 μm below the pia, propagated in two directions (Fig. 5f, right), and generated a passive current source in the superficial layers (Fig. 5f, middle). Moreover, in all animals (*n* = 8) this second current sink was abolished by local injection of baclofen in L2/3 (from 0.50 ± 0.12 to 0.00 ± 0.05 mV, *p* < 0.05, Wilcoxon signed rank test, Fig. 5g) while the first current sink did not significantly change (from 0.73 ± 0.12 to 0.43 ± 0.07 mV, *p* > 0.05, Wilcoxon signed rank test, Supplementary Fig. 7). Lastly, the surface positive potentials passively generated by the second current sink were clearly detectable with the surface electrode array (Fig. 6a, b). Many of these surface potentials were as large as those generated by the first current sink which was presumably due to synaptic input (Fig. 6b, c), although the average amplitude of the second current sink was smaller than the first (first: 0.71 ± 0.01 vs second: 0.59 ± 0.02 mV, *n* = 6 rats, *p* < 0.001, Wilcoxon rank sum test). Moreover, the second surface spike was significantly wider than the first (*p* < 0.05, Wilcoxon signed rank test, *n* = 6 animals, Fig. 6d, left) and importantly, optogenetically evoked surface potentials had a similar width (*p* > 0.05, Wilcoxon rank sum test, Fig. 6d, left). The peak latency of the second spike was also similar to that of the optogenetically evoked surface potential (*p* > 0.05, Wilcoxon rank sum test, Fig. 6d, right). Two-photon Ca^2+^ imaging of individual dendrites of L5 pyramidal cells (Fig. 7a–c) and simultaneous recording of surface potentials further revealed the strong correlation between the amplitude of the second surface potential and the peak fluorescence change (*r* = 0.7693, *p* < 0.01, *n* = 38 dendrites from 4 animals, Fig. 7d, e). To examine the predictive power of our characterization, we developed a simple classifier using half of the paired measurements of two-photon dendritic Ca^2+^ fluorescence and simultaneously recorded surface potentials (the “training” data); then we tested the classifier with the remaining half (the “validation” data, Fig. 7f, see Methods section for more details). Simply taking the average surface potential 50–60 ms after stimulus onset and setting a lower threshold θ_1_ that corresponds to the upper limit of fluorescence noise (mean M_0_ + one standard deviation SD_0_ when surface potentials are zero), the classifier predicts that the surface potentials correspond to Ca^2+^ spikes with 73.9% (34/46) accuracy (light gray area in Fig. 7f). With a higher threshold θ_3_ that corresponds to M_0_ + 3SD_0_, the classifier predicts Ca^2+^ spikes with 100% (10/10) accuracy (dark gray area in Fig. 7f). Taken together, we conclude that sensory stimulation evokes EPSPs that can be detected ~ 10 ms after the stimulation followed in some cases by dendritic Ca^2+^ spikes in L5 pyramidal neurons that have a comparable impact at the cortical surface in the form of positive potentials arriving ~ 50–60 ms after stimulation.

## Discussion

The contribution of the present study is threefold. Firstly we developed a novel optogenetic method to evoke dendritic Ca^2+^ spikes in vivo (Fig. 1a). This method enabled us to measure the contribution of dendritic Ca^2+^ spikes uncontaminated with synaptic inputs. Previous studies that reported the correlation between Ca^2+^ events and extracellular potentials^10, 37, 38^ did not separate these events; therefore it was unclear to which extent intrinsic dendritic Ca^2+^ currents contributed to the evoked potential. Secondly using the new method we found that dendritic Ca^2+^ spikes generate a passive current source in L1 (Fig. 1f) and therefore evoke a positive potential at the cortical surface with an inverted polarity relative to the expected dendritic sink at the site of generation of the dendritic spike (Fig. 3b). To our knowledge, these findings have not been reported in the literature. Lastly we quantified the relative contribution of dendritic Ca^2+^ spikes to the surface potential and showed that their contribution is on average equal to the contribution of synchronous, stimulus-evoked synaptic inputs (Fig. 6b, c).

Several types of current could in principle contribute to the late sensory-evoked sink including dendritic Ca^2+^ spikes, EPSPs generated by feedback input to the upper layers^39–41^, bAPs or NMDA spikes in the distal tuft^26^. Our data suggest that the late dendritic sink is due primarily or perhaps entirely to dendritic Ca^2+^ spikes. There were four observations that lead to this conclusion: firstly, the late sink in the upper layers was all-or-none in character. Second, the late sink was not due to synaptic input. Third, the late sink strongly correlated with dendritic Ca^2+^. Fourth, the late sink was abolished by baclofen. We have previously shown that baclofen abolishes dendritic Ca^2+^ spikes but does not block EPSPs or bAPs^12, 24^. We also previously found that local application of baclofen to L2/3 does not change the subthreshold input to L5 pyramidal neurons^25^ suggesting that network effects due to baclofen are negligible. Moreover, in the experiments using sensory stimuli in this study, the baclofen had no effect on the first sink while abolishing the late sink suggesting that the first sink was due to synaptic inputs that were unaffected by baclofen whereas the second sink was due to a non-synaptic process.

Both EPSPs and single or low-frequency bAPs have a comparably small effect on intracellular Ca^2+^ in the distal apical dendrite^22^. It is generally considered that the contribution of synaptic input to surface potentials is heavily dependent on the synchronicity of the synaptic inputs^42^. With a triggered sensory stimulation, the first signal is likely to arise from a barrage of synchronous synaptic inputs generated by the stimulus. However, the second signal ~ 200 ms after sensory stimulation has been shown to be the result of feedback^40^ that is more likely to be due to synaptic input that is less synchronous^41^. In our extracellular recordings, the late sink had a distinctly all-or-none character (unlike the late signal recorded intracellularly^40^) implying that the late synaptic input is more or less invisible in the LFP unless it reaches threshold for a postsynaptic event like a dendritic Ca^2+^ spike. On the other hand, the fact that the late sink we observed, when visible, had the same amplitude as the first sink, implies that the Ca^2+^ spike produces extracellular signals of similar magnitude to very synchronous synaptic inputs.

NMDA spikes are another form of postsynaptic dendritic spikes. However, they are normally confined to individual tuft branches^26, 43, 44^ whereas the late sink we recorded spread from depths that correspond to the location of the Ca^2+^ spike initiation zone in L5 pyramidal neurons^8^. Although the data suggest that the late extracellular sink is primarily due to dendritic Ca^2+^ spikes which represent a high conductance across a large area of membrane, it is still possible that the initiation of these spikes requires some combination of EPSPs, bAPs and/or NMDA spikes^18, 21, 26, 41^ that are each likely to have a smaller impact on the extracellular potential.

**Fig. 8:**
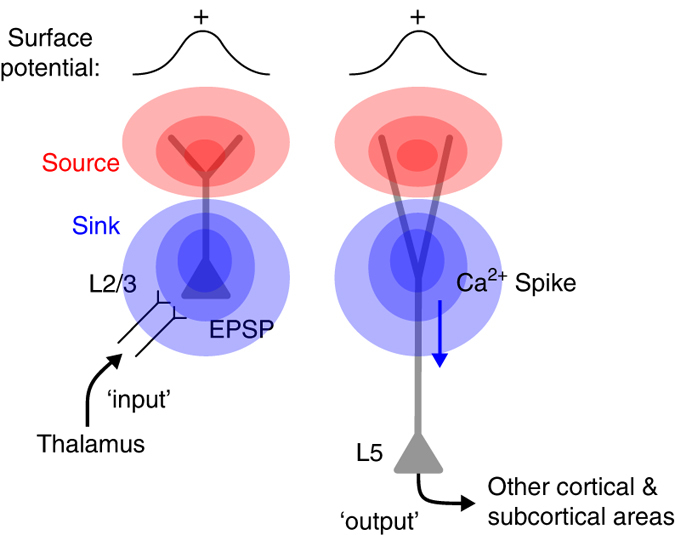
Diagram of the possible cellular mechanisms underlying surface positive potentials. The conventional view of surface positive potentials is that they are generated primarily by synaptic inputs from other cortical and subcortical regions to L2/3 pyramidal neurons through L4 (*left*)^51, 52^. We found that dendritic Ca^2+^ spikes of L5 pyramidal neurons (that lead to burst firing of these cells) could also generate the surface positive potentials (*right*)

The conventional view is that the surface positive potential primarily reflects a current source passively generated by a current sink corresponding to EPSPs in pyramidal neurons arriving in L2/3 (Fig. 8, left)^4^. Our study demonstrates that dendritic Ca^2+^ spikes in L5 pyramidal neurons can also evoke surface positive potentials that are as large as those evoked by EPSPs (Fig. 8, right). The corollary of this finding is that surface potentials evoked by dendritic Ca^2+^ spikes are an indication that activity has been recruited specifically in these cells. Furthermore, the Ca^2+^ spike is tightly linked to burst firing in these neurons^8, 19^. This is of central importance for understanding brain activity because L5 pyramidal neurons constitute the major output cells of the cerebral cortex. Because dendritic Ca^2+^ spikes are more easily evoked in the awake compared to the anesthetized brain^11^, a significant number of surface positive potentials in the awake brain recorded with conventional EEG are likely to represent dendritic Ca^2+^ spikes--that is, the output of a given cortical area. It should be noted that the surface potentials we characterized here were evoked by optogenetic or sensory stimuli. In principle, surface potentials could also be used to detect spontaneously occurring dendritic Ca^2+^ spikes (i.e., without sensory stimulation). In this case, the predictive power of this approach would be improved by first characterizing the surface potential resulting from evoked potentials.

In summary, we have developed a new method for optogenetically evoking dendritic Ca^2+^ spikes while recording LFPs, surface potentials, and Ca^2+^ fluorescence. We showed that these optogenetically evoked dendritic Ca^2+^ spikes can be detected at the cortical surface as positive potentials and that sensory-evoked potentials can also exhibit the same characteristics. We conclude that L5 pyramidal neurons are not simple passive dipoles but exhibit a more complex, dynamically changing laminar profile of current sinks and sources due to dendritic Ca^2+^ spikes. These results have strong implications for the interpretation of surface potentials that till now have not taken dendritic Ca^2+^ spikes into consideration.

## Methods

All experiments and procedures were approved and conducted in accordance with the guidelines given by the veterinary office of Landesamt für Gesundheit und Soziales Berlin.

### Virus injection

Female Wistar rats (P21–23) were initially anesthetized with isoflurane (~ 2% in O_2_, vol/vol, Abbott) before ketamine/xylazine anesthesia (75/10 mg per kg of body weight, respectively) was administered intraperitoneally and lidocaine (1%, wt/vol, Braun) was injected around the surgical site. Body temperature was maintained at ~ 36 °C by a heating pad and the depth of anesthesia was monitored throughout virus injection. Once anesthetized, the head was stabilized in a stereotaxic instrument (SR-5R, Narishige, Tokyo). The skull was exposed by a skin incision and a small hole (~ 0.5 × 0.5 mm^2^) was made above the hindlimb area of the primary somatosensory cortex (1.5 mm posterior to bregma and 2.2 mm from midline). AAV1.CamKIIa.hChR2(H134R)-eYFP.WPRE.hGH (Addgene 26969 P) or AAV1.CamKII.GCaMP6f.WPRE.SV40 purchased from the University of Pennsylvania Viral Vector Core was backloaded into a micropipette (Drummond) and was slowly injected (at 20 nl per min, total amount 40–50 nl) to L5. The pipette remained there for another 5 min after injection. The skin was sutured after retracting the pipette.

### Micro-periscope and optical stimulation

As shown in Supplementary Fig. 1, a 0.18 × 0.18 mm^2^ micro right-angled prism (Edmund Optics), a 100 μm core multi-mode optical fiber (NA 0.22, Edmund Optics), a custom-designed Grin lens (NA 0.2, outer diameter 0.25 mm), were assembled in-house using a UV curable adhesive (Noland). The other end of the optical fiber was coupled with a blue LED (peak wavelength 470 nm, Cree). The fiber was held and positioned by a stereotaxic micromanipulator (SM-15R, Narishige).

Optical stimulation of L5 through the micro-periscope was controlled by Power1401 and Spike 2 software (CED) and synchronized with the neural recording system via TTL signals. The duration of optical stimulation started at 10 ms and, if necessary, increased up to 30 ms to maximize the probability that dendritic Ca^2+^ spikes occurred. The resulting probability with the highest light intensity (12 mW/mm^2^) ranged from ~ 30 to ~ 100%.

### Extracellular recordings

Animals were initially anesthetized by isoflurane (~ 2% in O_2_, vol/vol, Abbott) before urethane anesthesia (0.05 mg per kg of body weight) in anesthetized experiments or ketamine/xylazine anesthesia (75/10 mg per kg of body weight, respectively) in awake experiments was administered intraperitoneally and lidocaine (1%, wt/vol, Braun) was injected around the surgical site. Body temperature was maintained at ~ 36 °C by a heating pad and the depth of anesthesia was monitored throughout experiment. Once anesthetized, the head was stabilized in the stereotaxic instrument and the skull was exposed by a skin incision. A ~ 1.5 × 1.5 mm^2^ craniotomy was made above the hindlimb area of the primary somatosensory cortex and the dura matter was removed. The exact location of the hindlimb area was mapped using the intrinsic optical imaging^45^ or flavoprotein autofluorescence imaging technique^46^. The area was kept moist with rat ringer for the entire experiment (135 mM NaCl, 5.4 mM KCl, 1.8 mM CaCl_2_, 1 mM MgCl_2_, 5 mM HEPES). In LFP recordings a linear array of 16 electrodes (NeuroNexus, A1 × 16-3 mm-100-177-A16 or A1 × 16-5 mm-100-177-A16) was perpendicularly inserted into the area such that the uppermost electrode was positioned at 100 μm below the pia. In surface potential recordings a 4 by 4 surface electrode array (NeuroNexus, E16-500-5-200-H16) was placed above the cortical area. In awake experiments the craniotomy was covered by silicone (Kwik-Cast, WPI), and the micro-periscope and the surface electrode were fixed to the skull with dental cement. After recovery the animal was placed in a rectangular arena (60 cm × 40 cm) and was free to move thereafter. Electrical activity was bandpass-filtered at 1–9 K Hz, digitized at 10 K Hz, amplified by ERP-27 system and Cheetah software (Neuralynx). LFPs were obtained by low-pass filtering the activity with the cutoff frequency of 300 Hz. Multi-unit activity was measured with a single tungsten electrode (~ 0.5 MOhm) and extracted through a bandpass filter (300–3 KHz).

### Principal component analysis (PCA) of LFP waveforms

Inspired by the commonly used PCA-based spike sorting technique^47^, LFP waveforms from 30 to 60 ms after stimulus onset were mapped in two dimensional space using the first and second principal components. Each dot in Figs. 1e, 4d represents a single waveform. Clusters were selected to visualize distinct features of waveform; unselected waveforms, e.g., ones between ‘ + ‘ and ‘o’ symbols show a mixed feature of the two types.

### Ca^2+^ fluorescence imaging with a micro-periscope

As shown in Supplementary Fig. 1, the imaging setup consisted of a micro-periscope, an LED (peak wavelength 535 nm, Cree), an excitation filter (555/20 bandpass, AHF), an emission filter (605/55 bandpass, AHF), a dichroic mirror (cutoff wavelength: 565 nm, AHF), a 80 × 80 pixel high-speed CCD camera with frame rate of 125 Hz (Redshirt Imaging), a 10 × infinity corrected objective (58–372, Edmund Optics), and a tube lens (Optem, RL091301-1). The calcium indicator Cal-590 AM (AAT Bioquest) was backloaded into a micropipette (Drummond) and slowly injected (at 20 nl per min, total 40–50 nl) to L5 (~ 1200 μm below the pia) of the hindlimb area 1.5–2 h before imaging experiments. The pipette remained there for at least 5 min after injection. The injection pipette was angled such that spillover dye, if any, would not be loaded in neurons in upper layers of the hindlimb area. Δ*F*/*F* was calculated as (*F*–*F*_*0*_)/*F*_*0*_, where *F* is the fluorescence intensity at any time point and *F*_*0*_ is the average intensity over the prestimulus period for 100 ms. The eYFP fluorescence upon excitation was measured before loading the calcium indicator and was subtracted from Δ*F*/*F*.

### Two-photon Ca^2+^ imaging and simultaneous surface recordings

Ca^2+^ fluorescence was measured using a custom-built two-photon microscope with B-scope (Thorlabs, Verginia), a water immersion objective (Olympus XLUMPlanFl, 0.95 NA) and a titanium sapphire laser (880 to 920 nm, 140-fs pulse width, Coherent Chameleon). Animals were either transfected with AAV1.CamKII.GCaMP6f.WPRE.SV40 or injected with Cal-520 AM (AAT Bioquest) in L5 as described in the previous sections. A craniotomy (diameter ~ 3 mm) was made under Urethane-anesthesia over the hindlimb area of the primary somatosensory cortex and the dura matter was removed. To simultaneously record surface potential, a glass electrode (0.5–1 MOhm) was placed under the cover glass (diameter 3 mm) such that the tip was at the location where the maximum sensory response was observed in the intrinsic or flavoprotein autofluorescence imaging. The cover glass and the recording pipette were glued to the skull. Ca^2+^ fluorescence was measured at a frequency of 100.30 Hz (142 × 512 pixels) by a GaAsP photomultiplier tube (Hamamatsu Photonics, Japan). Δ*F*/*F* was calculated as (*F*-*F*_*0*_)/*F*_*0*_, where *F* is the fluorescence intensity at any time point and *F*_*0*_ is the average intensity over the prestimulus three data points (duration 29.91 ms).

### Designing a simple classifier

Correlated (*r* > 0.8) paired measurements of two-photon dendritic Δ*F*/*F* and surface potential averaged over 50–60 ms were halved into the training and the validation data (90 measurements for each). Fluorescence noise was defined as M_0_ + SD_0_ where M_0_ and SD_0_ are, respectively, the mean and the standard deviation of the fluorescence change when surface potentials were zero. A lower threshold θ_1_ was derived from the intersection of the horizontal line (M_0_ + SD_0_) with the line fitted to the training data. A higher threshold θ_3_ was derived from the intersection of the line (M_0_ + 3SD_0_) with the fitted line. The performance of the classifier was measured using the validation data set.

### Drug application and sensory stimulation

All drugs used in this study were prepared on the day of experiment, backloaded to a micropipette (Drummond) and slowly injected (at 20 nl per min, total 40–50 nl) to their target cortical layer. The pipette was angled to avoid damaging the dendrites under study.

Sensory stimulation was provided by a single short electrical pulse (1 ms, 100 V) given to the contralateral hindpaw through a pair of conductive adhesive strips (Skintact).

### Data analysis and statistical methods

Analyses were conducted using Matlab (Mathworks). In optogenetic experiments current source density (CSD) analysis was performed on the average of all 100 evoked potentials at 16 cortical depths. In sensory stimulation experiments 100 measurements were divided into two groups, with or without the late sink; the late sink was considered present if the peak exceeded 3 × s.d. of the prestimulus activity. Instead of the original CSD method^48^, inverse CSD method^49, 50^ was used due to its significant advantages. Principal component analysis was performed on all LFP waveforms recorded at 600 μm below the pia and only the first and second principal components were used for cluster analysis.

Unless otherwise stated all values are indicated as mean ± s.e.m. and significance was determined by two-tailed, paired *t* tests or Wilcoxon tests at a significance level of 0.05. Each statistical test was chosen based on the data distribution using histograms. No statistical method was used to predetermine sample sizes, but our sample sizes are similar to those generally employed in the field. The variance was generally similar between groups under comparison. No blinding/randomization was performed.

### Data availability

The data that support the findings of this study are available from the corresponding author upon request.

## Electronic supplementary material

Supplementary Information
