# Supplementary material for: Dendritic calcium spikes are clearly detectable at the cortical surface
Source: Nat Commun. 2017 Aug 17;8:276. doi: 10.1038/s41467-017-00282-4 (PMC5561206; doi:10.1038/s41467-017-00282-4)
Supplement: Supplementary file 1 — Supplementary Information [file 41467_2017_282_MOESM1_ESM.pdf]

File Name: Supplementary Information
Description: Supplementary Figures.

Supplementary Figure 1

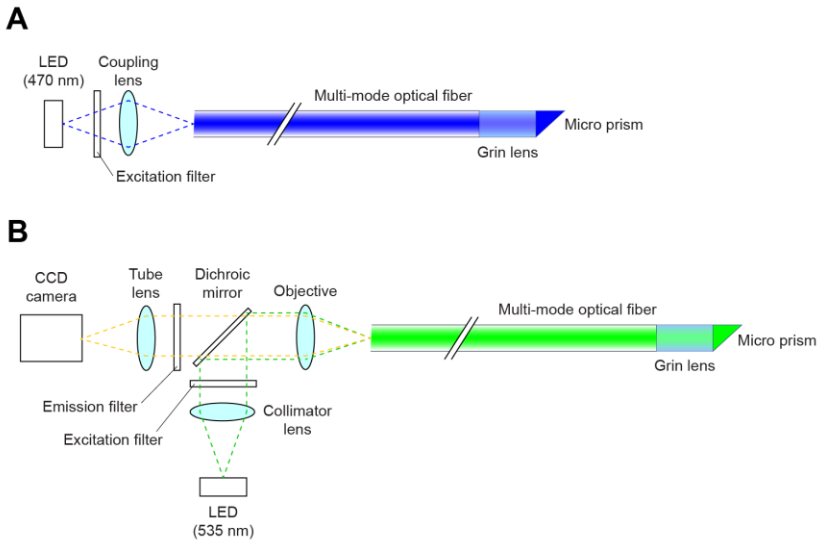

The micro-periscope setup for stimulation (A) and for Ca^{2+} fluorescence imaging (B).

Supplementary Figure 2

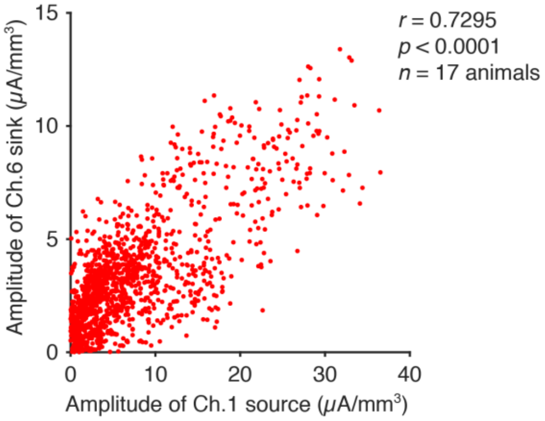

Strong correlation between middle layer sink (at channel 6) and superficial layer source (at channel 1).

Supplementary Figure 3

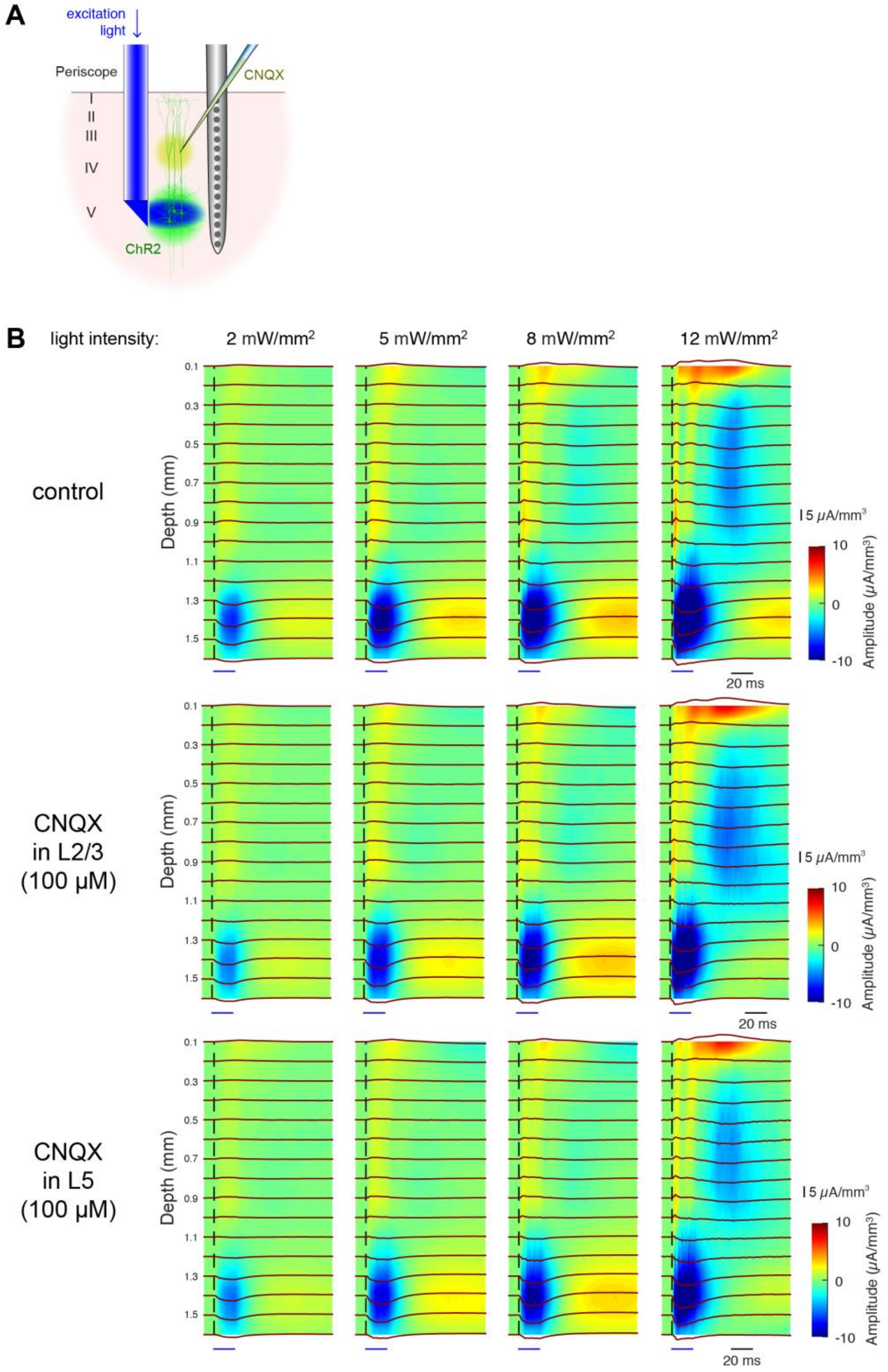

Optogenetically-evoked late sink was not impaired by local injection of 100 μM CNQX in L2/3 and L5. (**A**) Schematic diagram of the experiment. (**B**) CSD profiles in control (top) and drug conditions with different light intensities indicated at the top.

Supplementary Figure 4

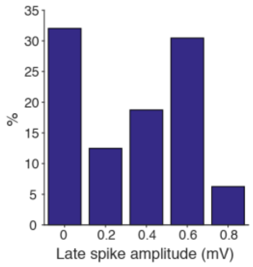

Bimodal distribution of late-spike amplitudes. Averaged over 100 measurements from one animal with the highest light intensity (12 mW/mm^2). Data from all animals (n = 17) show a bimodal distribution while the peak amplitude of detectable spikes and the fraction of undetectable spikes vary. See Fig. 1G for the distribution of detectable spike amplitudes.

Supplementary Figure 5

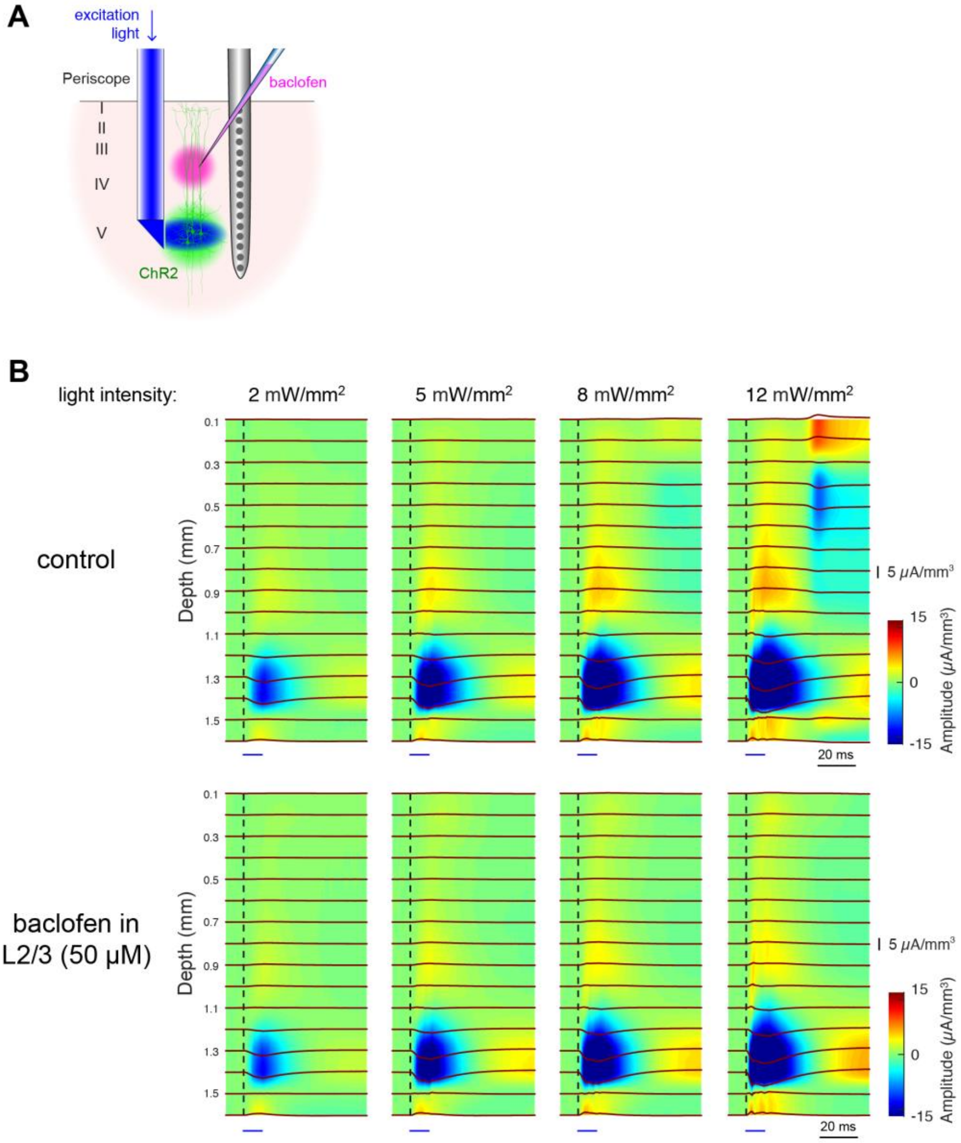

Optogenetically-evoked late sink was abolished by local injection of 50 µM baclofen in L2/3. (**A**) Schematic diagram of the experiment. (**B**) CSD profiles in control (top) and drug conditions (bottom) with different light intensities indicated at the top.

Supplementary Figure 6

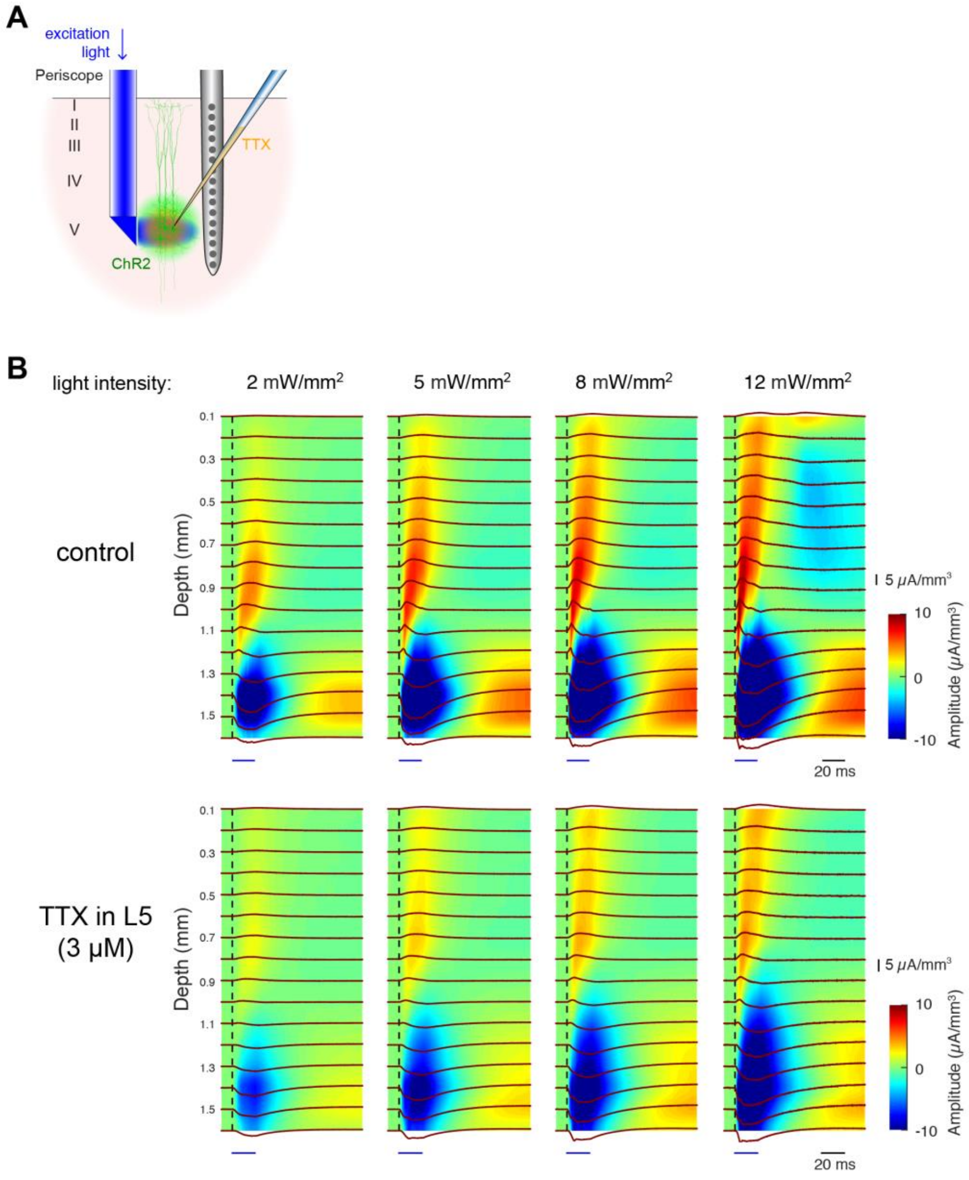

Optogenetically-evoked late sink was abolished by local injection of 3 μM TTX in L5. (**A**) Schematic diagram of the experiment. (**B**) CSD profiles in control (top) and drug conditions (bottom) with different light intensities indicated at the top.

Supplementary Figure 7

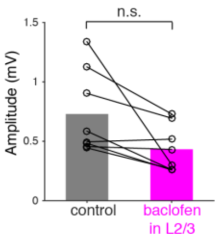

Sensory-evoked first sink was not impaired by local injection of 50 µM baclofen in L2/3 (*n* = 8, *p* > 0.05, Wilcoxon signed rank test).
